# Diagnosing cardiac disease during pregnancy: imaging modalities

**DOI:** 10.5830/CVJA-2016-022

**Published:** 2016

**Authors:** Ntobeko AB Ntusi, Petronella Samuels, Sulaiman Moosa, Ana O Mocumbi

**Affiliations:** Division of Cardiology, Department of Medicine, University of Cape Town and Groote Schuur Hospital, Cape Town, South Africa; Cape University Body Imaging Centre, Faculty of Health Sciences, University of Cape Town, Cape Town, South Africa; Military Hospital, Wynberg, Cape Town, South Africa; Instituto Nacional de Saúde and Department of Medicine, University Eduardo Mondlane, Maputo, Mozambique

**Keywords:** medical imaging, pregnancy, cardiovascular disease, X-ray, echocardiography, computed tomography, cardiovascular magnetic resonance, nuclear cardiology

## Abstract

Pregnant women with known or suspected cardiovascular disease (CVD) often require cardiovascular imaging during pregnancy. The accepted maximum limit of ionising radiation exposure to the foetus during pregnancy is a cumulative dose of 5 rad. Concerns related to imaging modalities that involve ionising radiation include teratogenesis, mutagenesis and childhood malignancy. Importantly, no single imaging study approaches this cautionary dose of 5 rad (50 mSv or 50 mGy). Diagnostic imaging procedures that may be used in pregnancy include chest radiography, fluoroscopy, echocardiography, invasive angiography, cardiovascular computed tomography, computed tomographic pulmonary angiography, cardiovascular magnetic resonance (CMR) and nuclear techniques.

Echocardiography and CMR appear to be completely safe in pregnancy and are not associated with any adverse foetal effects, provided there are no general contra-indications to MR imaging. Concerns related to safety of imaging tests must be balanced against the importance of accurate diagnosis and thorough assessment of the pathological condition. Decisions about imaging in pregnancy are premised on understanding the physiology of pregnancy, understanding basic concepts of ionising radiation, the clinical manifestations of existent CVD in pregnancy and features of new CVD. The cardiologist/physician must understand the indications for and limitations of, and the potential harmful effects of each test during pregnancy. Current evidence suggests that a single cardiovascular radiological study during pregnancy is safe and should be undertaken at all times when clinically justified. In this article, the different imaging modalities are reviewed in terms of how they work, how safe they are and what their clinical utility in pregnancy is. Furthermore, the safety of contrast agents in pregnancy is also reviewed.

## Abstract

Pregnant women with known cardiovascular disease (CVD) or a newly diagnosed cardiac condition in pregnancy often require cardiovascular imaging during the pregnancy to confirm the diagnosis, to assess disease severity and stratify risk, to prognosticate, to plan for appropriate management and to assess response to therapy (Table 1). A variety of cardiovascular imaging modalities are available for such purposes and include X-ray [which encompasses chest radiography, cardiovascular computed tomography (CCT), computed tomographic pulmonary angiography (CTPA), coronary computed tomographic angiography (CCTA), fluoroscopy and invasive angiography], echocardiography, cardiovascular magnetic resonance (CMR) and nuclear techniques.

**Table 1:** Rationale for use and indications for imaging of CVD in pregnancy

Evaluation of biventricular structure, size and function
Evaluation of native and prosthetic valve disease
Evaluation of pregnancy-induced hypertension and hypertensive heart failure of pregnancy
Evaluation of congenital heart disease
Evaluation of myocarditis
Evaluation of specific cardiomyopathies
• Dilated cardiomyopathy
• Peripartum cardiomyopathy
• Hypertrophic cardiomyopathy
• Arrhythmogenic right ventricular cardiomyopathy
• Iron-overload cardiomyopathy
• Restrictive cardiomyopathy
• Myocardial infiltration (e.g. sarcoidosis)
• Left ventricular non-compaction
• Systemic rheumatic diseases (e.g. rheumatoid arthritis, systemic lupus erythematosus, systemic sclerosis)
• Other less-common diseases (e.g. Chagas disease, Churg-Strauss syndrome)
Evaluation of pericardial disease
• Pericarditis
• Pericardial effusions
• Pericardial tumours
• Pericardial effusive-constrictive syndrome
• Pericardial constriction
Evaluation of great vessels and pulmonary veins
Evaluation of cardiac masses (differentiation of tumour from thrombus)
Evaluation of infective endocarditis
Evaluation of ischaemic heart disease
• Diagnosis of myocardial infarction and its sequelae
• Assessment of myocardial viability
• Assessment for inducible ischaemia
• Coronary imaging
• Assessment of suspected coronary artery fistula
• Assessment of suspected anomalous coronary origins
Differentiation of ischaemic versus non-ischaemic cardiomyopathy
Evaluation of mechanical dyssynchrony
Evaluation of unexplained heart failure or stroke

Of these, diagnostic X-ray and nuclear procedures emerge as the greatest source of concern for patients and clinicians alike. However, most diagnostic radiological procedures do not expose the pregnant woman to a degree of radiation that would threaten the well-being of the developing pre-embryo, embryo or foetus.^1^ Furthermore, as cardiological imaging focuses mainly on the chest, there is minimal direct exposure of the lower abdomen, where the baby may lie within the main X-ray beam; hence radiation doses to the developing foetus tend to be small.^2^ The mechanisms of action, safety and clinical utility of various cardiovascular imaging modalities in pregnancy are considered below.

We performed a systematic search of the published literature on cardiovascular imaging in pregnancy, published in the English language, through PUBMED (January 1966 to December 2015), OVID, Cochrane Database of Systematic Reviews and hand search of reference lists from selected articles. All search engines were searched using the key words: ‘pregnancy’, ‘cardiovascular imaging’, ‘echocardiography’, ‘X-ray’, ‘angiography’, ‘fluoroscopy’, ‘computerised tomography’, ‘cardiovascular magnetic resonance’ and ‘nuclear cardiology’. Articles with important insights about cardiovascular imaging in pregnancy are included.

## Ionising radiation and pregnancy

Ionising radiation refers to electromagnetic radiation produced by X-ray equipment, the radioactive isotopes (radionuclides) used for radiation therapy (Table 2). The accepted cumulative dose of ionising radiation during pregnancy is 5 rad (which is also equal to 50 mSv or 50 mGy),^3^ and no single diagnostic study exceeds this maximum. For example, the amount of exposure to the foetus from a two-view chest X-ray of the mother is only 0.00007 rad.^4^

**Table 2:** Measures of ionising radiation

*Measure*	*Definition*	*Conventional units*	*SI units*
Exposure	Number of ions produced by X-rays per kg of air	Roentgen (R)	Coulombs/kg (C/kg)
Absorbed dose	Amount of energy deposited per kg of tissue	Radiation absorbed dose (rad)	Gray (Gy)
			1 Gy = 100 rad
KERMA	Kinetic energy released per unit mass	Radiation-absorbed dose (rad)	Gray (Gy)
			1 Gy = 100 rad
Dose equivalent	A measure of radiation-specific biological damage in humans	Roentgen equivalents man (rem)	Sievert (Sv)
			1 Sv = 100 rem
Relative effective dose	Amount of energy deposited per kg of tissue normalised for biological effectiveness	Roentgen equivalents man (rem)	Sievert (Sv)
		(1 rem = 1 rad for X-rays)	1 Sv = 100 rem
			(1 Sv = 100 rad for X-rays)
Activity	Amount of radioactivity expressed as the nuclear transformation rate	Curie (Ci)	Bequerel (Bq)
			1 Ci = 3.7 × 10^10^ Bq

Possible deleterious effects of ionising radiation include (1) genetic consequences, the risks of which can be assessed only from animal studies; (2) carcinogenesis, which can be assessed from survivors of nuclear bombings and patients exposed for medical reasons; and (3) teratogenic effects on the developing embryo or foetus.^2^ Most cardiovascular diagnostic procedures expose the embryo and foetus to less than 50 mSv,^5^ which does not increase reproductive risks (either birth defects or miscarriage).^6^

The reported dose of radiation with consequent increased incidence of birth defects or miscarriage is above 200 mSv.^7^ Termination of pregnancy on the grounds of ionising radiation exposure is not recommended unless there is sufficient documentation that the estimated foetal dose exceeds 15 rad (150 mSv).^8^

An important determinant of the consequence of radiation exposure in pregnancy is the stage in which the radiation exposure occurs.^1^,^9^ In the first two weeks following conception or the second two weeks from the last menstrual period, the developing embryo is resistant to the malforming effects of X-rays. However, the developing embryo is sensitive to the lethal effects of X-rays, although doses much higher than 5 rad (50 mSv) are necessary to cause a miscarriage.^10^ From the third to the eighth week of pregnancy, in the period of early embryonic development, the embryo is hardly affected, in terms of birth defects, pregnancy loss, or growth retardation, unless the exposure is substantially above 200 mSv.^11^ From the eighth to the 15th week of pregnancy, the embryo or foetus is sensitive to the effects of radiation, particularly on the central nervous system (CNS). However, for the development of microcephaly and other CNS malformations, the radiation exposure has to be sufficiently high. The threshold for an observed effect on intelligence quotient is estimated to be greater than 30 rad (300 mSv).^12^

Cardiovascular diagnostic studies do not reach these levels and, therefore, these effects are rarely of concern for patients. The most sensitive period for CNS teratogenesis is between eight and 15 weeks of gestation, therefore non-urgent radiological testing should be avoided during this time. Rare consequences of prenatal radiation exposure include a slight increase in the incidence of childhood leukaemia and, possibly, a small change in the frequency of genetic mutations.^13^ Such exposure is not an indication for pregnancy termination, however.

Appropriate counselling of patients before radiological studies are performed is critical. After 20 weeks of gestation, the foetus is fully developed and it again becomes resistant to the effects of radiation exposure. At this late stage, there is no evidence of increased risk of birth defects or miscarriage from radiological diagnostic studies.^14^

## Deleterious effects of ionising radiation

## Radiation-induced teratogenesis

CNS malformations, in particular microcephaly and mental retardation, are the most commonly seen non-stochastic complications following high-dose radiation exposure. Following Hiroshima, many Japanese bomb victims who were exposed *in utero* to doses greater than 10 to 150 rad developed microcephaly.^15^ A linear, dose-related association between severe mental retardation and radiation was also found, with the important caveat that most cases followed exposure during weeks eight to 15 of gestation.^16^,^17^

## Radiation-induced malignancy

Exposure to as little as 1 or 2 rad has been associated with an increase in childhood malignancies, especially leukaemia, occurring in a stochastic fashion.^13^ For example, the background rate of leukaemia in children is about 3.6 per 10 000.^18^ Exposure to 1 or 2 rad increases this rate to five per 10 000.^19^ While these doses do fall within the range of some radiographic studies, the absolute increase of risk (~ 1 in 10 000) is very small.^20^ Therefore, physicians should carefully weigh the risks and benefits of any radiographic study and include the mother in the decisionmaking process whenever possible.

## Radiation-induced mutagenesis

Radiation can cause germ-line mutations, potentially affecting future generations. Although radiation is commonly believed to create bizarre new mutations, data show that it usually merely increases the frequency of mutations occurring naturally in the general population.^21^ The dosage required to double this baseline mutation rate is between 50 and 100 rad, far more than the radiation doses occurring in common cardiovascular radiographic studies.^22^

The most important factor for physicians to remember is that the currently accepted maximum limit of ionising radiation exposure to the foetus during pregnancy is a cumulative dose of 5 rad (50 mSv or 50 mGy).^3^,^10^,^20^,^23^

## Non-ionising radiation and pregnancy

The reproductive risk of non-ionising radiation, which includes electromagnetic fields from computers, microwave ovens, microwave communication systems, cellular phones, power lines, household appliances, heating pads and warming blankets, airport metal screening devices and diagnostic ultrasound has been studied extensively. Two national committees of scientists in the US evaluated the risk from these non-ionising radiation sources. The first report was published in 1993 from the Oak Ridge Associated University panel^24^ created by the White House, while the second was the product of the committee of the National Academy of Sciences.^25^ Both of these groups concluded that the reproductive risk of non-ionising radiation is minimal, if even existent.^24^,^25^

## Chest radiography

The chest X-ray is the most commonly performed diagnostic cardiovascular radiographic examination, and is able to produce accurate images of the heart, lungs, airways, blood vessels and the bones of the spine and chest. The chest X-ray utilises small amounts of radiation (0.00002 to 0.00007 rad)^4^,^9^ when a focused beam of radiation is passed through the body, resulting in a black-and-white image recorded on special film or a computer.

X-rays are able to differentiate tissues in the body because of varying densities (each tissue allows a different amount of radiation to pass through and expose the X-ray-sensitive film).^26^ Dense bone absorbs much of the radiation while soft tissue, such as heart muscle, allows more of the X-rays to pass through. Consequently bones appear white on the X-ray, soft tissue shows up in shades of grey and air appears black.

**Fig. 1.**
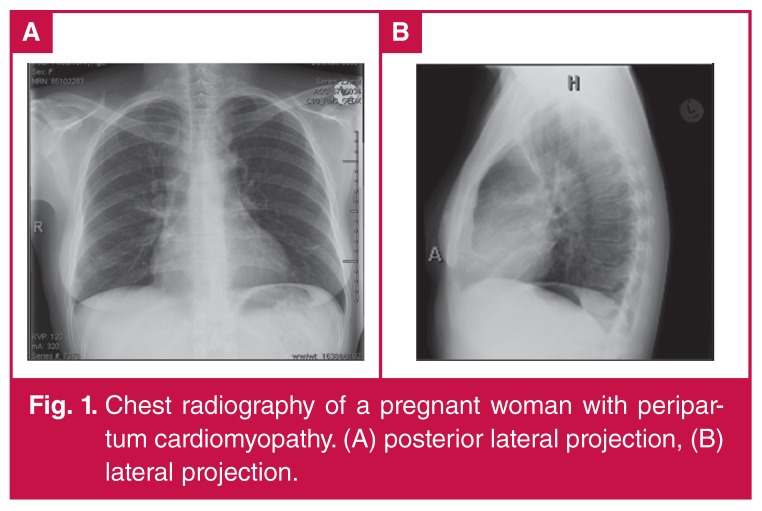
Chest radiography of a pregnant woman with peripartum cardiomyopathy. (A) posterior lateral projection, (B) lateral projection.

Medically indicated diagnostic chest radiographic studies can be safely performed in pregnancy (Fig. 1), provided the equipment works properly and the abdomen of the patient is adequately shielded. The risk of not making the diagnosis often far surpasses the risk of radiation in such instances.^27^

## Fluoroscopy and invasive angiography

Fluoroscopy is a type of medical imaging that shows a continuous X-ray image on a monitor, much like an X-ray movie. Fluoroscopy is routinely used to screen suspected stuck prosthetic valves and for percutaneous transvenous mitral commissurotomy (PTMC) in those with symptomatic mitral stenosis in pregnancy. Furthermore, fluoroscopy is the basis for imaging during invasive angiographic procedures (including coronary angiography and haemodynamic studies). There are many situations where the benefit of performing these procedures is much greater than any small possible harm that might arise from radiation exposure.^20^ For a typical fluoroscopic study, the amount of radiation occurring is in the range of 0.001 to 0.05 rad; dosage depends on duration of fluoroscopic time.^28^

As always with any medical exposure, each particular procedure must be clinically justified, including taking into account when the procedure needs to occur and the anticipated radiation dose to the foetus. Once justified, due diligence is taken to optimise when and how the procedure is performed to minimise radiation exposure to the foetus, consistent with achieving the desired clinical outcome. The radiation exposure to the foetus predominantly arises from scattered radiation within the patient.^20^,^29^

Some of the main methods for minimising the dose to the foetus include: (1) restricting the X-ray beam size to as small as is necessary; (2) choosing the direction of the primary beam so that it is as far away from the foetus as possible; (3) ensuring that the overall exposure time is as short as possible; (4) selecting appropriate exposure factors; (5) calculating the dose by a knowledgeable medical physicist, if there is concern; and (6) using a lead apron on the table to shield any primary beam from the X-ray tube reaching the foetus (Table 3).

**Table 3:** Approaches to minimising foetal radiation during A B cardiovascular imaging in pregnancy

Restricting the X-ray beam size to as small as is necessary
Choosing the direction of the primary beam so that it is as far away from the foetus as possible
Ensuring that the overall exposure time is as short as possible
Selecting appropriate exposure factors
Defer abdominal examinations if possible; imaging examinations of the thorax are associated with negligible risks to the conceptus
Whenever possible, ultrasound is the preferred modality for abdominal imaging in pregnancy
Magnetic resonance imaging is emerging as an alternative in centres where it is widely available
Using a lead apron on the table to shield any primary beam from the X-ray tube reaching the foetus
Calculations of dose by a knowledgeable medical physicist if there is concern
The radiation dose should be kept as low as reasonably achievable (ALARA principle)

## Echocardiography

Echocardiography is an imaging modality that uses highfrequency (2–10 MHz) sound waves to image cardiac structures and to give reproducible information about cardiac structure and function. Ultrasound is produced when a piezo-electric crystal, mounted in a transducer, is stimulated by an electric current.^30^

Ultrasound waves are not audible and are harmless to tissue at the intensities used in diagnostic imaging. The passage of sound waves depends on the acoustic impedance of tissues. Most ultrasound waves pass through tissues to deeper structures further from the surface, but reflected sound returns to strike the crystal, deforming it and producing electric signals, which correspond to the degree of deformation.^30^ This electrical information is transformed so it can be displayed on a cathoderay tube as pulses of light. Due to the speed of sound within the body being relatively constant, the depth of the tissue interface can be calculated, and reflected echoes are displayed on the screen on a depth scale.^31^

Blood reflects little sound and appears relatively black/hypoechoic compared with the myocardium, which reflects more of the ultrasound and appears relatively white/hyperechoic. The heart valves are even more echogenic. Neither bone nor air is a good transmission medium for ultrasound waves; therefore as the heart is surrounded by lung and the bony cage of the thoracic cavity, the ultrasound beam must be aimed through specific gaps, known as acoustic windows (e.g. parasternal, apical, subcostal and suprasternal), to produce images of the heart and vasculature.^31^ Given the lack of ionising radiation, echocardiography is an attractive first-line investigation for most forms of CVD encountered in pregnancy

**Fig. 2.**
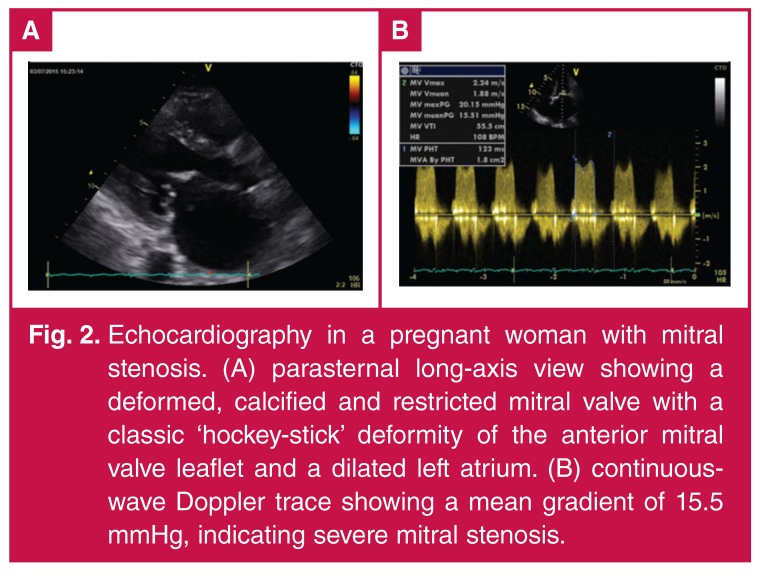
The process of Confidential Enquiry into Maternal Deaths.

M-mode and two-dimensional (2D) echocardiography provide real-time imaging of heart structures throughout the cardiac cycle; more recently, three-dimensional (3D) echocardiography has been developed.^32^ Doppler echocardiography provides information on blood movement inside cardiac structures and on the haemodynamics^33^ (Fig. 2). Tissue Doppler imaging (TDI) provides information about movement of cardiac structures.^33^ The relationship between the dynamics of cardiac structures and the haemodynamics of the blood inside these structures provides information about cardiac diastolic and systolic function.^33^ Echocardiography is continuously evolving and constantly being augmented by newer modalities, such as tissue harmonics, speckle tracking, tissue Doppler strain and tissue characterisation.^34^

To date, there have been no reports of documented adverse foetal effects from diagnostic ultrasound procedures, including duplex Doppler imaging.^1^ There are no contra-indications to echocardiography during pregnancy, and ultrasound is preferred over X-ray as the primary method of foetal imaging during pregnancy.^35^ Energy exposure from ultrasonography has been arbitrarily limited to 94 mW/cm^2^ by the US Food and Drug Administration (FDA).^36^

Doppler and colour echocardiographic scans work by concentrating a beam of sound in a small area, and therefore can cause heating of local tissues if held in the same place for a long time.^37^ Most scans automatically reduce the power of the ultrasound beam when Doppler is used, to decrease the intensity. Nowadays most echocardiographic machines have a low thermal index and so pose very little risk. Dobutamine is favoured in pregnancy over adenosine for stress echocardiography.^38^

## Cardiovascular computed tomography (CCT)

Computed tomography (CT) is a diagnostic imaging procedure that uses X-rays to demonstrate cross-sectional images of the body acquired in different orthogonal planes. The crosssections or slices are reconstructed from the measurements of attenuation coefficients of X-ray beams in the volume of the object studied.^39^ The fundamental principle of CT is premised on tissue density traversed by the X-ray beam, which can be calculated from the attenuation coefficient. In other words, CT permits reconstruction of tissue density by 2D sections perpendicular to the axis of the acquisition system. Unlike X-ray radiography, the detectors of the CT scanner do not produce an image, but rather measure the transmission of a thin beam (1–10 mm) of X-rays through a full scan of the body, and the image of that section is taken from different angles, allowing retrieval of information on the depth of the tissues imaged.^40^

Complex mathematical algorithms are used to construct an image from the raw data; a typical CT image is composed of 512 rows, each of 512 pixels, i.e. a square matrix of 512 × 512 = 262 144 pixels (one for each voxel). A typical CCT study gives 0.06 to 0.09 rad.^41^ Similarly, the effective radiation dose for CT pulmonary angiogram (CTPA) protocols is generally between 2.2 and 7 mSv (0.02–0.07 rad).^42^

Often, for CCT and CTPA, the imaging field of view includes the lungs and breasts; the radiation dose can be reduced by shielding, lowering the peak kilovoltage or lowering the tube current.^43^ In general, lower-dose protocols result in images with poor resolution and greater noise, therefore reductions must consider image quality and diagnostic confidence.^44^

CTPA remains the imaging modality of choice for diagnosis of pulmonary embolism in pregnancy and is preferred for its general superiority over ventilation-perfusion scintigraphy.^45^ Ventilation-perfusion scintigraphy may be indeterminate in up to 25% of patients imaged in pregnancy.^46^ In addition, the foetal radiation dose from CTPA is substantially less than that from ventilation-perfusion scintigraphy in all trimesters, even if halfdose perfusion-only scintigraphy is used.^47^,^48^

## Cardiovascular magnetic resonance

CMR is a remarkably powerful imaging modality, free of ionising radiation, with high spatial and temporal resolution, performed via excitation of hydrogen protons within a powerful magnetic field.^49^ The strong magnetic field aligns the nuclear magnetisation spin of the hydrogen protons, which are then excited by radiofrequency (RF) pulses (pulse sequences). After the RF pulses are switched off, the protons give off energy as they precess back to their equilibrium magnetisation; this dissipated energy is detected by the MR receiver coils. Fourier transformation is then used to convert frequencies into images.

The signal from a given tissue (e.g. heart muscle) is determined by the proton density (PD) and by two specific relaxation parameters: longitudinal relaxation time (T1) and transverse relaxation time (T2).^49^ PD, T1 and T2 vary substantially for different tissues, and may vary substantially within the same tissue from health to disease; these differences are used to generate contrast in MR images.^50^ To prevent artifacts from cardiac motion, CMR images are generated with fast sequences gated to the R wave of the electrocardiogram. Respiratory motion may be eliminated by acquiring CMR images in end-expiratory breath-hold.

**Fig. 3.**
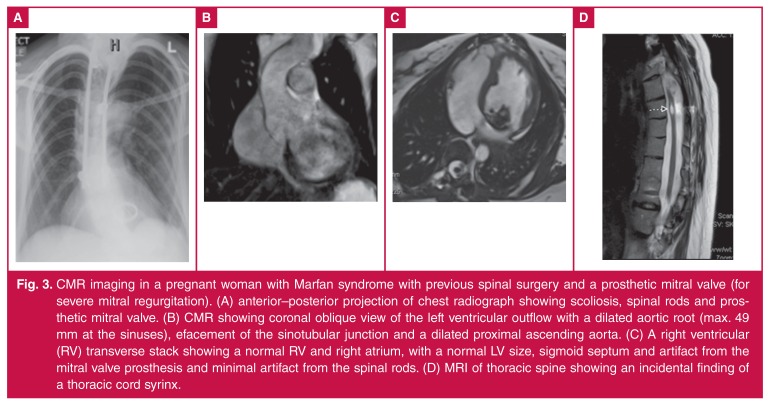
CMR imaging in a pregnant woman with Marfan syndrome with previous spinal surgery and a prosthetic mitral valve (for severe mitral regurgitation). (A) anterior–posterior projection of chest radiograph showing scoliosis, spinal rods and prosthetic mitral valve. (B) CMR showing coronal oblique view of the left ventricular outflow with a dilated aortic root (max. 49 mm at the sinuses), efacement of the sinotubular junction and a dilated proximal ascending aorta. (C) A right ventricular (RV) transverse stack showing a normal RV and right atrium, with a normal LV size, sigmoid septum and artifact from the mitral valve prosthesis and minimal artifact from the spinal rods. (D) MRI of thoracic spine showing an incidental finding of a thoracic cord syrinx.

MR has been used to evaluate obstetric, placental and foetal abnormalities in pregnant patients for more than 25 years. MR imaging is recognised as a beneficial diagnostic tool and is utilised routinely to assess multiple conditions that affect the pregnant patient (Fig. 3) as well as the foetus. To date, there has been a paucity of systematic studies directed towards determining the relative safety of using MR procedures in pregnant patients.^51^ There has been no evidence of harm from the use of CMR and other forms of MR imaging in pregnancy.^51^

Safety concerns include possible bio-effects of the static magnetic field of the MR system, risks associated with exposure to the gradient magnetic fields, the potential adverse effects of RF energy, possible adverse effects related to heating and to the combination of these three electromagnetic fields, possible acoustic injury from the vibration and noise in the scanner, and possible toxicity from gadolinium-based contrast agents used in patients with renal dysfunction.^52^ MR environment-related risks are difficult to assess for pregnant patients due to the number of possible permutations of the various factors that are present in this setting (e.g. differences in field strengths, pulse sequences, exposure times).

However, several experimental and clinical investigations of the effects of MR in pregnancy showed no evidence of injury or harm to the foetus or the mother.^53^,^54^ Even the few human studies performed in pregnant human subjects exposed to MR imaging or the MR environment have not reported adverse outcomes for the subjects.^55^,^56^ In recent times, there has been increasing concern that acoustic noise associated with MR may impact on the foetus; however this remains unproven in recent large studies.^57^

In summary, CMR up to 3T appears to be safe in all stages of pregnancy.^58^ Higher field strengths have not been evaluated in the setting of pregnancy. CMR, where available, together with echocardiography, remains preferable to any studies using ionising radiation for cardiovascular imaging in pregnancy, in particular during the first trimester. Despite the lack of harm from MR in pregnancy, the current guidelines of the FDA require labelling of MR devices to indicate that the safety of MRI with regard to the foetus ‘has not been established’.

## Nuclear cardiovascular imaging

Diagnostic nuclear medicine investigations also involve ionising radiation. Unlike X-rays, nuclear techniques involve the inhalation, ingestion or injection of a small quantity of a radioactive isotope bound in a substance that targets a particular organ, for example the heart. The gamma radiation emitted by the radioactive isotope is detected outside the body by electronic receptors of a gamma camera, which displays images or functional data about the heart.^59^

The most commonly used radioisotope, technetium-99m (^99m^Tc), is a metastable daughter product following negative beta decay of molybdenum-99. ^99m^Tc decays to ^99^Tc with a halflife of six hours, releasing a mono-energetic gamma photon of 140 keV.^60^

Nuclear studies that may be performed during pregnancy include ventilation-perfusion scintigraphy for diagnosis of pulmonary embolism, myocardial perfusion imaging where ^99m^Tc may be combined with several compounds that localise to active myocardial cells, allowing ischaemic areas of the heart to be determined, and, less commonly, cardiac ventriculography where ^99m^Tc can be used to evaluate cardiac function (ejection fraction) by imaging the ventricles. The dose of radiation passed on to the foetus during a ventilation-perfusion scan is about 0.05 rad.^61^

Along with conventional gamma scintigraphic imaging, the two major nuclear imaging techniques are positron-emission tomography (PET) and single photon-emission computed tomography (SPECT). Both imaging modalities are now standard in the major nuclear medicine services.

PET is based on the principle of positron annihilation by using radionuclides that decay through positive beta decay.^62^ Positrons generated by the decay combine with an electron and annihilate, releasing two photons, with energies of 0.51 MeV, in the process. The photons are released in opposite directions.

The most commonly used compound for PET imaging is fluoro-2-deoxyglucose (^18^FDG), which is initially metabolised within the cell, is unable to progress to the citric acid cycle, and is not easily excreted by the cell.^62^ Hence, cells that have a high glucose metabolism concentrate, ^18^FDG, can then be imaged. The sections are reconstructed by algorithms, similar to but more complex than those used for conventional CT, to accommodate the 3D acquisition geometries.^63^ Correction by considering the physical phenomena provides an image representative of the distribution of the tracer within the heart. In PET scanning, an effective dose of the order of 8 mSv is delivered to the patient.^64^

SPECT imaging is based on detectors that rotate around the patient to obtain a digital representation of a 3D radioactive distribution of the chest. The injected radioactive tracers emit during their disintegration, gamma photons, which are detected by an external detector after passing through the surrounding tissue.^65^ In SPECT, the main radioactive isotopes are ^99m^Tc, iodine and thallium-201 (which is used primarily for studies on the heart). To increase the sensitivity and resolution of SPECT systems, converging channel collimators were developed.^66^

Both PET and SPECT benefit from electrocardiographic gating used to enhance tomographic myocardial scintigraphy.Therefore, the radioactivity from the myocardium and the electrical activity of the heart are coupled. Depending on the procedure, the mother and baby will generally receive a small radiation dose with SPECT. It is unlikely that any diagnostic nuclear medicine investigation would result in the radiation dose of the foetus approaching 20 mGy. It is ideal that radioactive isotopes are avoided during pregnancy. However, if there is a real clinical need for such imaging to be performed, the risk to the mother and foetus is minimal.^20^

## Contrast agents

A variety of oral and intravascular contrast agents are used with X-ray and MR procedures. Radiopaque agents used with CT and conventional radiography contain derivatives of iodine and have not been studied comprehensively in human pregnancy. However, iohexol, iopamidol, iothalamate, ioversol, ioxaglate and metrizamide have been studied in animals and do not appear to be teratogenic.^67^

Neonatal hypothyroidism has been associated with some iodinated agents taken during pregnancy.^68^ Therefore iodinebased contrast agents are relatively contra-indicated in pregnancy, unless absolutely essential for a correct diagnosis. Studies requiring views before and after the administration of contrast agents will necessarily have greater radiation exposure. While most contrast agents pass into the breast milk, they have not been associated with problems in nursing babies.^67^ Despite *in vitro* concerns, iodinated contrast agents seem safe to use in pregnancy.^69^ Radioactive isotopes of iodine are mutagenic and are absolutely contra-indicated during the pregnancy.^70^

Paramagnetic contrast agents used during CMR have not been studied systematically in pregnant women. Animal studies have demonstrated increased rates of spontaneous abortion, skeletal abnormalities, and visceral abnormalities when given at two to seven times the recommended human dose.^71^ It is not clear whether gadolinium-based contrast agents are excreted into human breast milk. It is important to emphasise that gadolinium-based contrast agents have not been associated with any harm in human pregnancy.^72^,^73^

The 2007 American College of Radiology (ACR) guidance for safe MR practices (expanded and updated in 2013) recommends that intravenous gadolinium should be avoided in pregnancy and should only be used if absolutely essential, until there is further information about these agents.^74^,^75^ Consequently, the FDA has classified gadolinium as a category C drug, meaning it can be considered in pregnancy ‘if the potential benefits justify the potential risks to the fetus’.

## Safety counselling

When a pregnant mother considers any radiation exposure, the most prominent question in her mind is likely to be, ‘Is this safe for my baby?’ To answer this question, the physician must carefully choose words that will help a patient understand the real, although very small, risks of exposure. The general population’s total risk of spontaneous abortion, major malformations, mental retardation and childhood malignancy is approximately 286 per 1 000 deliveries. Exposing a foetus to 0.50 rad adds only about 0.17 cases per 1 000 deliveries to this baseline rate, or about one additional case in 6 000.^6^ Such numbers often do not make much sense to patients, and it is incumbent on the clinician to take the time to allay fears, ensuring good and clear communication during counselling.

‘Safe’ is a relative term but one that physicians should not be afraid to use. When a radiographic study is needed for appropriate management of a pregnant patient, the ACR recommends that ‘health care workers should tell patients that X-rays are safe and provide patients with a clear explanation of the benefits of X-ray examinations.’^74^ One tool that physicians may consider using to reassure patients is Table 3, which compares the dosage of radiation provided by various common diagnostic studies with the accepted limit of 5 rad (50 mSv). A patient’s particular study could also be plotted on this graph, showing the clear margin of safety that exists for all single diagnostic studies.

## Conclusion

Pregnant women with known or suspected CVD often require cardiovascular imaging. The accepted maximum limit of ionising radiation exposure to the foetus during pregnancy is a cumulative dose of 5 rad (50 mSv or 50 mGy). Concerns related to imaging modalities that involve ionising radiation include teratogenesis, mutagenesis and childhood malignancy. Importantly, no single imaging study approaches this cautionary dose of 5 rad (Table 4). Elective studies may be deferred until the pregnancy is over or the gestational period is beyond 20 weeks, and there are several strategies that may be employed to minimise radiation to the foetus (Table 3). Echocardiography and CMR appear to be completely safe in pregnancy and are not associated with any adverse foetal effects, provided there are no general contraindications to MR imaging. Current evidence suggests that a single cardiovascular radiological study during pregnancy is safe and should be undertaken at all times when clinically justified.

**Table 4:** Doses to the foetus from radiological and nuclear medicine examinations

*Examination*	*Estimated foetal dose(mGy)*
Chest radiograph	< 0.0001
Pulmonary CTA	0.01–0.66
CCTA (prospective gating)	1.0
CCTA (retrospective gating)	3.0
Abdominopelvic CTA	6.7–56.0
Direct fluoroscopy (groin to heart catheter passage)	0.094–0.244 mGy/min
Coronary angiography	0.074
Electrophysiological procedures	0.0023–0.012 mGy/min
Lung perfusion	0.6
Lung ventilation	0.005–0.09
Myocardial perfusion	5.3–17
Gated blood pool	6.0
PET viability	6.3–8.1
PET perfusion	2.0
Maximum recommended dose	5 rad or 50 mGy

As a general guide, centres where medical imaging is performed should have signs to remind mothers to notify the staff if they may be pregnant. The potential risks of each imaging modality must be discussed with the mother before she undergoes such imaging. Pregnant women must be made to understand that exposure from a single diagnostic procedure does not result in harmful foetal effects. Specifically, exposure to less than 5 rad has not been associated with an increase in foetal anomalies or pregnancy loss. Therefore concerns about possible effects of high-dose ionising radiation exposure should not prevent medically indicated diagnostic X-ray procedures from being performed on pregnant women. Consultation with an expert in dosimetry calculation may be helpful in calculating estimated foetal dose when multiple diagnostic X-ray procedures are performed in pregnancy.

In general, the radiation safety principle, ALARA (as low as reasonably achievable), minimising radiation and release of radioactive materials should be employed at all times. In addition, the use of iodine-based contrast agents for X-ray, fluoroscopy and CT scanning, and the use of gadolinium-based contrast agents for CMR are safe in pregnancy and should be used when the potential benefit justifies the potential risk to the foetus. However, the use of radioactive isotopes of iodine is contra-indicated for therapeutic use during pregnancy.
